# Motor and Nonmotor Symptoms of Parkinson's Disease: Antagonistic Pleiotropy Phenomena Derived from *α*-Synuclein Evolvability?

**DOI:** 10.1155/2018/5789424

**Published:** 2018-11-22

**Authors:** Yoshiki Takamatsu, Masayo Fujita, Gilbert J. Ho, Ryoko Wada, Shuei Sugama, Takato Takenouchi, Masaaki Waragai, Eliezer Masliah, Makoto Hashimoto

**Affiliations:** ^1^Tokyo Metropolitan Institute of Medical Science, 2-1-6 Kamikitazawa, Setagaya-ku, Tokyo, Japan; ^2^PCND Neuroscience Research Institute, Poway, CA, USA; ^3^Department of Physiology, Nippon Medical School, Tokyo, Japan; ^4^Institute of Agrobiological Sciences, National Agriculture and Food Research Organization, Tsukuba, Ibaraki, Japan; ^5^Department of Neuroscience, National Institute on Aging, Bethesda, MD 20892, USA

## Abstract

Lewy body diseases, such as Parkinson's disease (PD), dementia with Lewy bodies (DLB), and multiple system atrophy (MSA), are associated with a wide range of nonmotor symptoms (NMS), including cognitive impairment, depression and anxiety, sleep disorders, gastrointestinal symptoms, and autonomic failure. The reason why such diverse and disabling NMS have not been weeded out but have persisted across evolution is unknown. As such, one possibility would be that the NMS might be somehow beneficial during development and/or reproductive stages, a possibility consistent with our recent view as to the evolvability of amyloidogenic proteins (APs) such as *α*-synuclein (*α*S) and amyloid-*β* (A*β*) in the brain. Based on the heterogeneity of protofibrillar AP forms in terms of structure and cytotoxicity, we recently proposed that APs might act as vehicles to deliver information regarding diverse internal and environmental stressors. Also, we defined evolvability to be an epigenetic phenomenon whereby APs are transgenerationally transmitted from parents to offspring to cope with future brain stressors in the offspring, likely benefitting the offspring. In this context, the main objective is to discuss whether NMS might be relevant to evolvability. According to this view, information regarding NMS may be transgenerationally transmitted by heterogeneous APs to offspring, preventing or attenuating the stresses related to such symptoms. On the other hand, NMS associated with Lewy body pathology might manifest through an aging-associated antagonistic pleiotropy mechanism. Given that NMS are not only specific to Lewy body diseases but also displayed in other disorders, including amyotrophic lateral sclerosis (ALS) and Huntington's disease (HD), these conditions might share common mechanisms related to evolvability. This might give insight into novel therapy strategies based on antagonistic pleiotropy rather than on individual NMS from which to develop disease-modifying therapies.

## 1. Introduction

It is well established that synucleinopathies, including PD, DLB, and MSA, are characterized by a number of NMS, such as cognitive impairment, depression and anxiety, sleep difficulties, gastrointestinal disturbance, and autonomic failure. Because some NMS occur in the prodromal disease stages, NMS are both mechanistically and therapeutically important [1, 2]. Recently, in this field, there has been great interest in better understanding NMS, a topic which has been prominently reviewed [3–9]. Nevertheless, the mechanisms which underlie NMS in neurodegenerative diseases remain obscure.

Accordingly, the main objective of this paper is to discuss how NMS might be involved in the pathogenesis of synucleinopathies and related disorders. Given that a variety of NMS often occur during the course of multiple neurodegenerative conditions, it is predicted that NMS might be triggered by multiple pathologic factors, including protein aggregation and inflammation. One possibility then would be that NMS might be passive phenomena as a result of neurodegeneration. Yet, an alternative and nonmutually exclusive possibility is that NMS might be a consequence of evolvability [10], whereby NMS information might be transgenerationally delivered to offspring encoded in APs, such as *α*S and A*β*, perhaps preventing the stresses relevant to NMS in offspring. On the other hand, NMS may manifest as symptoms of aging-associated neurodegenerative disease through an antagonistic pleiotropy mechanism in the parental brains. Finally, we propose that a better understanding of this hypothetical view would facilitate development of a therapy strategy against NMS in synucleinopathies.

## 2. Motor and Nonmotor Symptoms in Synucleinopathies

In PD and related synucleinopathies, treating motor signs and symptoms due to the degeneration of dopaminergic neurons in the substantia nigra has long been the focus of disease management. However, in recent years, because of increased clinical recognition and relevance to patient life quality, the nonmotor aspects of such disorders have attracted increasing interest. Clinically, NMS consists of four domains: neuropsychiatric (e.g., depression, anxiety, apathy, hallucinations, and dementia), autonomic (e.g., constipation, orthostatic hypotension, urinary changes, and sweating abnormalities), sleep (e.g., insomnia, sleep fragmentation, excessive daytime sleepiness, rapid eye movement, sleep disorder, and restless leg syndrome), and sensory dysfunction (e.g., pain and olfactory dysfunction) [11–13]. Such diversity of NMS may be consistent with the widespread distribution of *α*S pathology in the gut [14, 15] as well as brainstem and neocortex in PD brain [16], in which multiple populations of aminergic neurons may be affected, including serotonergic and noradrenergic neurons. Thus, the classic Parkinsonian motor syndrome is now regarded as but one unitary symptom type among many disparate symptoms of the synucleinopathies.

## 3. Are NMS Passive Phenomena?

**Figure 1:**
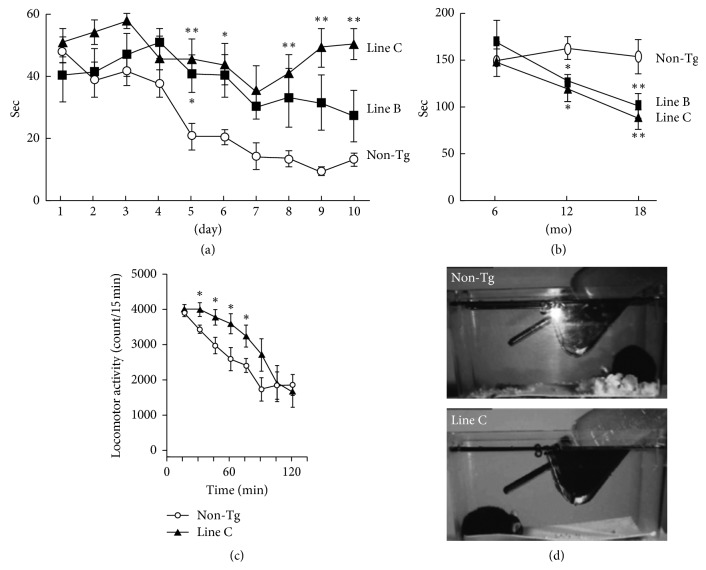
Altered behaviors observed in a DLB model mouse (a and b). Tg mice expressing DLB-linked P123H*β*S were characterized by memory disorder (∼6 month: by the water maze test) (a) and being more prominent than motor deficits (∼12 month: by the rotarod treadmill test) (b). See Reference [21] for the details. (c and d) The P123H*β*S mice exhibited depression-like behaviors as assessed from the results of the locomotor activity (6∼10 month) (c) and the nest building test (6∼10 month) (d). See Reference [21] for the details. Reprinted with permission from References [21, 22].

Overall, it would appear possible that similar to motor symptoms (MS), NMS might be passive phenomena in response to amyloid fibrils and inflammation during the progression of PD and other disorders, including ALS and HD [17–20]. Indeed, such a view is supported by the results of studies in animal models. For instance, we also observed that transgenic (Tg) mice expressing DLB-linked P123H*β*-synuclein (*β*S) developed progressive neurodegeneration, as characterized by axonal swelling, astrogliosis, and behavioural abnormalities. Interestingly, expression of the memory abnormality (∼6 months of age on water maze testing) was more prominent compared with the motor deficits (∼12 months of age on the rotarod treadmill test) [21] (Figures 1 and 1). Furthermore, P123H*β*S mice exhibited depression-like behaviors as assessed by locomotor activity (∼6 months) and the nest building test (∼6 months) [22] (Figures 1 and 1). Collectively, this suggested that motor deficits were preceded by NMS, such as memory dysfunction and depression-like features. Similarly, hyperactivity and depression-like behaviors were observed in A53T*α*S Tg mice [23] and a tauopathy mouse model [24]. Since APs are constitutively expressed using artificial promoters, such as thy-1, prion promoter, and calmodulin kinase II*α*, in Tg mice models of neurodegenerative diseases [21, 23, 24], it is presumed that the accumulation of protofibrillar APs, including P123H*β*S, *α*S, and tau, may interfere with signal transduction and transcription, eventually leading to the manifestation of neurobehavioral phenotypes such as depression.

There, however, would seem to be little evolutionary advantage for the passive association of NMS with neurodegenerative diseases in aging. Distinct from other organisms, humans are characterized by an extended postmenopausal senescence due to stable nutritional supply and an absence of predators [25]. Although nature remains biologically indifferent to the human condition during postreproductive time of life, a recent study suggests that the “grandmother effect” in humans may be evolutionarily beneficial because nursing of their first grandchild by a grandmother is beneficial to their daughter to encourage birth of a second grandchild [26]. From this perspective, if NMS are simply passive phenomena following neurodegeneration features, such as accumulation of toxic aggregates of APs and inflammation during aging, this would be evolutionarily not advantageous and might have been selected out in evolution.

## 4. NMS as Active Phenomena Dependent on Evolvability and Antagonistic Pleiotropy

One might wonder as to why NMS have not been eliminated through natural selection. Indeed, it was recently described that both MS and NMS were observed in 1-methyl-4-phenyl-1, 2, 3, 6-tetrahydropyridine-treated marmosets, a nonhuman primate model [27]. Considering that NMS by themselves are rather consequences that are severely disabling for patients in aging and cannot transgenerationally be delivered to offspring, we predict that NMS might be linked to some physiologically beneficial effects during development and/or reproductive stages. Notably, such a view is reminiscent of the evolvability of APs such as *α*S and A*β* in the brain [10]. Based on the heterogeneity of protofibrillar forms of APs in terms of structure and cytotoxicity, we proposed that APs might act as vehicles to deliver information regarding diverse biological stressors [10]. Mechanistically, we speculate that *α*S, a monomer of which is unstable due to its intrinsically disordered nature [28], might become more stable through oligomerization, leading to formation of diverse strains of protofibrils. Such stable *α*S protofibrils may be feasible for transgenerational transmission to the offspring.

**Figure 2:**
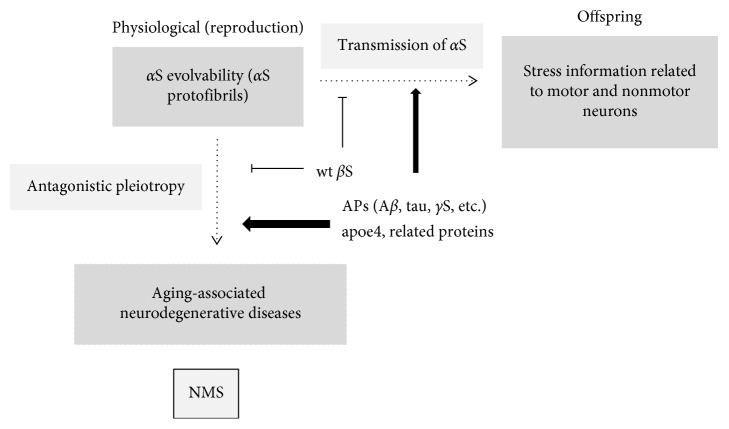
Schematics of the motor and nonmotor symptoms in neurodegenerative diseases and amyloid evolvability in the human brain. Hypothetically, stress information derived from both motor and nonmotor neurons might be integrated into diverse structures of αS protofibril strains that are transgenerationally transmitted to offspring during reproduction as a physiological phenomenon. On the other hand, the *α*S protofibrils may manifest as neurodegenerative disease associated with NMS through an antagonistic pleiotropy mechanism during the postreproductive senescent period. Both processes are stimulated by various proteins, including A*β*, tau, *γ*S, and apoE, but are suppressed by wild-type *β*S.

In this way, information regarding both MS and NMS might be integrated into the evolvability of *α*S (Figure 2). Presuming that NMS-related information is transgenerationally transmitted to offspring through evolvability of *α*S, it would benefit offspring. Yet, on the other hand, *α*S aggregates may also cause neurodegenerative disease and associated NMS through an antagonistic pleiotropy mechanism during aging. Thus, evolvability would be an epigenetic phenomenon in which APs transgenerationally transmit such information to offspring to cope with future stressors affecting the offspring's brain. It is predicted that NMS might be active phenomena related to evolvability.

## 5. Modulation of NMS Evolvability by Other Factors

Because *α*S pathology is promoted by other APs, such as A*β* [29] and tau [30], it is likely that evolvability of these molecules might also positively affect the evolvability of *α*S (Figure 2). Furthermore, *β*S is also of particular interest because the evolvability of *α*S may be positively and negatively regulated by wild-type and mutant *β*S, respectively [21, 31]. Similarly, since *γ*-synuclein (*γ*S), the third member of the synuclein family of peptides [32, 33], may be involved in the regulation of *α*S evolvability because *γ*S is associated with neuritic pathology, such as in dystrophic neurites and spheroid structures, in the brains of sporadic cases of PD, DLB, and neurodegeneration with brain iron accumulation type 1 [34, 35]. Furthermore, it was shown that the formation of aggregates and deposits of *γ*S is facilitated after its oxidation at methionine 38 [36]. Collectively, it is possible that all synuclein family peptides might cooperate in NMS-related *α*S evolvability.

Moreover, aggregation of *α*S was also shown to be influenced by apolipoprotein E (apoE), a major Alzheimer's disease (AD) risk factor, with apoE4 having the most robust stimulatory effect compared with other isoforms (E2 and E3). Since apoE4 binds to A*β* and promotes fibrillization, we previously suggested that evolvability of A*β* might be enhanced by apoE4 [25]. Similarly, other apolipoproteins, such as ApoJ, and ApoA1, might also associate with *α*S to modify evolvability [37, 38]. Notably, the importance of membrane lipids, such as raft, in *α*-synucleinopathies has been previously described [39]. Thus, it is tempting to speculate that the pathological role of membranous functions in *α*-synucleinopathies in aging might reflect the regulation of *α*S evolvability by the membrane in development/reproduction.

In addition, there has been increasing interest in transgenerational epigenetic inheritance in which various epigenetic factors like DNA methylation, histone modifications, and regulatory RNAs have been described [40]. Therefore, it is possible that some of these epigenetic factors are involved in regulating *α*S evolvability (Figure 2).

## 6. Therapeutic Implication

Notably, some NMS such as cognitive and neuropsychiatric features, [1] as well as constipation and other gastrointestinal symptoms [2], often are expressed in the prodromal disease stage of neurodegeneration. Since recent studies suggest that disease-modifying therapy (DMT) for neurodegenerative diseases should be initiated at earlier stages, NMS may be important from both the mechanistic and therapeutic standpoints.

As discussed, NMS might be either passive phenomena during the course of neurodegeneration or an active phenomena derived from evolvability through antagonistic pleiotropy. In the former case, neuropathogenic factors, such as fibrils and inflammation, are presumed to be situated upstream of NMS. Although therapeutic strategies are thought to target those neuropathogenic factors, no DMT has thus proven effective in relieving NMS. Alternatively, each nonmotor symptom might be individually targeted. For instance, dysfunction of hypothalamic-pituitary-adrenal axis (HPA), a central regulatory system underlying stressors [41], has been implicated in contributing to depressed mood and anxiety, in patients with depression [42]. In this context, it was shown that deletion of corticotropin-releasing factor receptor type 1 (CRFR1) mitigated the amyloid-*β* pathology in a mouse model of AD, lending support to the notion that suppressing the HPA axis through CRFR1 antagonism may be an effective therapeutic strategy against AD [43]. Given that CRFR1 in the brain is involved in the regulation of endocrine, behavioural, autonomic, and visceral in response to stress [44], the suppression of CRFR1 signaling might also be effective for other neurodegenerative diseases with NMS conditions. Also, pharmacological approaches, such as NMDA antagonists and dopamine agonists might be effective for some NMS such as depression [45, 46].

Yet, if the alternate explanation is the case, more unconventional therapeutic strategies might be employed. For instance, in addition to targeting neuropathogenic factors, such as fibrils and inflammation, disease-modifying strategies would focus on antagonistic pleiotropy rather than on the individual NMS. Currently, the mechanism underlying antagonistic pleiotropy is unclear. In this regard, however, it is noteworthy that a recent study revealed pleiotropic associations of allelic variants in a 2q22 region with risks of major human diseases, such as vascular disease, cancer, and neurodegenerative disease, and mortality [47], suggesting a possibility that the serine/threonine TGF*β*/activin receptor-signaling pathways might be involved in the regulation of antagonistic pleiotropy. In support of this view, importance of the serine129 with phosphorylates *α*S has been well characterized in PD [48]. In particular, accumulation of *α*S serine129 phosphorylation in Lewy bodies is a hallmark of the pathogenesis in PD [49]. The similar is the case of tau in AD although involvement of both serine/threonine kinases and tyrosine kinase has been described [50, 51]. If this view is the case, modification of the TGF*β*/activin receptor-signaling pathways could be therapeutically effective for the entire symptoms, including both MS and NMS in neurodegenerative diseases and perhaps other aging-associated chronic diseases. Further investigations are warranted to test this intriguing possibility.

## 7. Conclusions

Although increasingly clear that NMS are important early biomarkers as well as targets for disease-modifying therapy for synucleinopathies, such as PD, DLB, and MSA, the mechanisms by which NMS are involved in the pathogenesis of the disease have not been fully understood. We hypothesized that stress information derived from both MS- and NMS-relevant neurons might be integrated into the diverse structures of αS protofibrils and are transgenerationally transmitted, which is probably beneficial to ward against forthcoming stressors in offspring, i.e., evolvability.

However, in parental brain, *α*S protofibrils might manifest later in life associated with aging-associated neurodegenerative disorders through the antagonistic pleiotropy mechanism. Therefore, our theory implies that NMS, because they are derived from the physiological phenomenon of evolvability, are not selected by evolution. It further introduces a new framework that antagonistic pleiotropy might be a valid therapeutic target for disease-associated NMS.

Although the concepts of amyloid evolvability and the antagonistic pleiotropy phenomena derived from amyloid-like proteins in neurodegenerative diseases are intriguing, such a theory requires further experimental validations and at present is far from explaining the complex pathophysiology of NMS in PD. Thus, further investigations are definitely warranted to demonstrate our hypothesis.
